# Plumbagin sensitizes leukemia cells to cisplatin by promoting oxidative stress, apoptosis, and DNA damage

**DOI:** 10.7150/ijms.124444

**Published:** 2026-05-29

**Authors:** Yan-Ning Chen, Jai-Wei Lee, Tsu-Ming Chien, Chia-Hung Yen, Bing-Hung Chen, Hsueh-Wei Chang

**Affiliations:** 1 Graduate Institute of Natural Products, Kaohsiung Medical University, Kaohsiung 80708, Taiwan.; 2 Department of Tropical Agriculture and International Cooperation, National Pingtung University of Science and Technology, Pingtung 912301, Taiwan.; 3 School of Post-Baccalaureate Medicine, Kaohsiung Medical University, Kaohsiung 80708, Taiwan.; 4 Department of Urology, Kaohsiung Medical University Hospital, Kaohsiung Medical University, Kaohsiung 80708, Taiwan.; 5 Department of Urology, Kaohsiung Gangshan Hospital, Kaohsiung Medical University, Kaohsiung 820111, Taiwan.; 6 Department of Biotechnology, College of Life Science, Kaohsiung Medical University, Kaohsiung 80708, Taiwan.; 7 Department of Biomedical Science and Environmental Biology, College of Life Science, Kaohsiung Medical University, Kaohsiung 80708, Taiwan.; 8 Department of Medical Research, Kaohsiung Medical University Hospital, Kaohsiung 80708, Taiwan.; 9 Center for Cancer Research and Research Center for Molecular Medicine, Kaohsiung Medical University, Kaohsiung 80708, Taiwan.; 10 Drug Development and Value Creation Research Center, Kaohsiung Medical University, Kaohsiung 80708, Taiwan.

**Keywords:** plumbagin, combined treatment, cisplatin, leukemia, oxidative stress

## Abstract

This study focused on validating the synergistic antiproliferative activity and mechanism of cisplatin/plumbagin (PLB) in leukemic cells versus normal macrophages. Leukemic cells (MOLT-4) treated with cisplatin/PLB (3 μM/0.4 μM) for 24 h showed markedly reduced viability (45.51%) compared to cisplatin or PLB (81.07% or 76.36%), while normal macrophages (NR8383) retained high viability in cisplatin/PLB (~93.15%). The enhanced cytotoxicity in leukemia cells was driven by oxidative stress: the general antioxidant *N*-acetylcysteine (NAC) and the mitochondrial ROS scavenger MitoTEMPO substantially reversed the combination effect. Cisplatin/PLB increased total ROS and mitochondrial superoxide in MOLT-4 cells more than in macrophages, and provoked loss of mitochondrial membrane potential and depletion of glutathione; these alterations were mitigated by NAC and MitoTEMPO. Oxidative stress led to higher apoptosis in leukemia cells than normal macrophages, shown by increased subG1 fraction (validated via NAC), higher annexin V positivity (validated via NAC and MitoTEMPO), elevated cleaved PARP and caspases-9/-3, Bax upregulation, and a reduced p-Bcl-2 (anti-apoptotic Ser70)/Bcl-2 ratio (validated via NAC), whereas caspase-8 changed only modestly. DNA damage markers (γH2AX and 8-OHdG) were also increased in MOLT-4 cells and attenuated by NAC and MitoTEMPO. Overall, cisplatin/PLB triggers selective and oxidative stress-dependent in leukemia cells while sparing normal macrophages, supporting the combination as a promising antileukemic approach with limited toxicity.

## Introduction

Leukemia is a type of hematological malignancy with the mass production characteristics of abnormal immature blood cells, blocking the generation of normal blood cells. There were 66,890 estimated new cases and 23,540 estimated deaths worldwide caused by leukemia in “Cancer statistics, 2025” 1. Males show a mildly higher number of cases of fatalities from leukemia than females. Generally, four common types of leukemia have been classified, i.e., acute myeloid leukemia (AML) 2, acute lymphoblastic leukemia (ALL) 3, chronic lymphocytic leukemia (CLL) 4, and chronic myeloid leukemia (CML) 5. ALL is the most common malignancy in children and teenagers 6, 7. Therefore, the present study focused on improving the inhibition of ALL cell proliferation.

Combined treatment is an improved anticancer strategy that combines two or more therapeutic agents 8. The ideal combined treatment is effective cancer therapy with fewer toxic side effects because it uses a low dose but generates synergistic effects. Leukemia therapy favors combined treatments, including chemotherapy, radiotherapy, targeted therapy, and bone marrow transplantation 9, 10. However, patients occasionally experience adverse effects from receiving the leukemia treatments 11. Investigating additional potential anti-leukemia drugs for combined treatment with clinical medications is necessary to alleviate side effects.

Cisplatin is the first-line anticancer drug for several cancers, such as oral, breast, bladder, testicular, ovarian, prostate, cervical, lung, leukemia, and others 12, 13. Although cisplatin generally has practical chemotherapeutic functions, its potential side effects limit cancer treatment 14. A combination of natural products with cisplatin has been reported in several anticancer studies 15, 16, thereby enhancing cisplatin sensitivity in cancer cells and reducing cytotoxicity in normal cells.

Natural products have made a significant contribution to anticancer drug discovery 17. Several natural products exhibit high antiproliferative efficacy against cancer cells with low cytotoxicity to normal cells 18, 19. For example, *Nepenthes* plants, such as *N. mirabilis, N. alata, N. khasiana,* and* N. rajah*, are commonly used as traditional herbs in Southeast Asia 20. In addition to the antibacterial, antifungal 21, and anti-inflammatory 22 effects, several *Nepenthes* extracts exhibit antiproliferative functions against cancer cells 23. For example, the ethyl acetate extract of N. thorelii x (ventricosa x maxima) (EANT) shows antiproliferative activity in leukemia cells 24. Plumbagin (PLB), the main bioactive compound (naphthoquinone) of EANT, showed antiproliferative effects on leukemia cells 24, cervical 25, and tongue 26 cancer cells. Furthermore, PLB induces apoptosis of CML (K562) 27. The safety of PLB was validated in normal peripheral blood mononuclear cells 28 and in animal studies 28.

Prooxidant drugs can suppress leukemia; for example, arsenic trioxide generates ROS that induce mitotic arrest and apoptosis in AML 29, while all-trans retinoic acid derivative elevates ROS to promote differentiation and inhibit leukemic cell growth 30. Because both PLB 24 and cisplatin 31 induce ROS in leukemia cells, their combination may further elevate ROS levels and enhance antileukemic activity. While cisplatin/PLB combined treatment has been described for cervical 25 and tongue 26 cancer cell models, it has not yet been reported in leukemia cells. Consequently, the potential antiproliferative effects of cisplatin/PLB on leukemia cells warrant a detailed assessment.

The present study aims to evaluate the antiproliferative effects and assess the anticancer mechanism of cisplatin/PLB on leukemia cells, particularly for ALL.

## Materials and Methods

### Cell cultures, viability, synergy index, and reagents

Two kinds of ATCC human T-cell ALL cell lines (Manassas) derived from leukemia were chosen, including MOLT-4 32 and J45.01. NR8383 33, the normal rat lung macrophage from ATCC, is regarded as the normal control cell. MOLT-4 is a primary cortical T-cell ALL line with intact CD45 expression and functional T-cell receptor signaling 34, while J45.01 is a CD45-deficient Jurkat mutant lacking this signaling 35. MOLT-4 and J45.01 cells were kept in RPMI medium (Gibco) and supplemented with 10% FBS and antibiotics. NR8383 cells were kept in F12 medium containing 15% FBS and antibiotics. ATP-lite reagents (PerkinElmer Life Sciences) were used to assess cell viability, according to the user instructions. Briefly, after removing the medium, 100 μL of serum-free medium and 50 μL of lysis buffer were added per well and shaken at 100 rpm for 5 min. Then 100 μL of lysate was transferred to a white 96-well plate, 12.5 μL of D-luciferin/luciferase substrate was added, and the plate was shaken at 100 rpm for 5 min. Finally, luminescence was measured with a luminometer 36. The synergy index (α value) for the combined cisplatin/PLB was calculated as previously described 37: α = (viability fraction of cisplatin × viability fraction of PLB) / viability fraction of cisplatin/PLB. Values of α = 1, > 1, and < 1 indicate additive, synergistic and antagonistic interactions, respectively.

PLB (Selleckchem) was dissolved in DMSO. The DMSO concentration in all experiments was the same (0.1%). *N*-acetylcysteine (NAC; 5 mM, 1 h) (Sigma-Aldrich) 38 and MitoTEMPO (20 μM, 1 h) (Cayman Chemical) 39, 40 were used as inhibitors for reactive oxygen species (ROS) and mitochondrial superoxide (MitoSOX). These inhibitors were pretreated to validate the role of ROS and MitoSOX in PLB treatments.

### ROS, MitoSOX, mitochondrial membrane potential (MMP), and glutathione (GSH) assay

According to the user's manual, flow cytometry reagents were applied to detect several oxidative stress-related indicators, i.e., dihydroethidium (DHE) (Sigma-Aldrich) 39 (5 μM for 30 min) for ROS, MitoSOX™ Red (Molecular Probes, Invitrogen, Eugene, OR, USA) 41 (50 nM for 30 min) for MitoSOX, JC-1 (Sigma-Aldrich) 42 (0.2 μM for 30 min) for MMP, and 5-chloromethylfluorescein diacetate (CMF-DA) (Thermo Fisher Scientific) 41 (0.1 μM, 20 min) for GSH detections. Finally, these oxidative stress-related responses were detected using flow cytometry analysis. Notably, JC-1 green appears in damaged MMP, while JC-1 red appears in healthy MMP. A high ratio (JC-1 green/JC-1 red) and GSH (-) proportion indicate the MMP and GSH depletion.

### Cell cycle assay

Cells were fixed with 75% ethanol overnight and stained with 7-aminoactinomycin D (7AAD) (Biotium) 19 (1 μg/mL, 30 min, 37 °C). After washing, cells were conducted with a flow cytometer (Guava easyCyte, Luminex, TX, USA).

### Annexin V-apoptosis assay

Annexin V can bind to phosphatidylserine in apoptotic cells. Reagents containing annexin V/7AAD kit (Strong Biotech) 19 were used for this double staining. After washing, cells were conducted with a flow cytometer.

### Western blotting for apoptotic signaling

Apoptotic signaling was detected using antibody sampler kits (#9915/#9941/#9942, Cell Signaling Technology), recognizing proapoptotic (Poly (ADP-ribose) polymerase (PARP), Bcl-2 associated X (Bax), and cleaved caspases 3/8/9) and anti-apoptotic proteins (B-cell lymphoma 2 (Bcl-2) and phosphorylated Bcl-2 (Ser70) (5H2) (anti-apoptotic p-Bcl-2 43, #2827)).

### γH2AX and 8-hydroxy-2′-deoxyguanosine (8-OHdG) assays

γH2AX primary antibody (Santa Cruz Biotechnology) was diluted to 500X. After washing, the fixed cells were incubated with the Alexa Fluor 488-secondary antibody (1:10000) (Jackson Laboratory). Without the secondary antibody step, the 8-OHdG-FITC antibody was diluted to 10000X (Santa Cruz Biotechnology) for mixing with cell suspensions. Both γH2AX and 8-OHdG were detected using flow cytometry analysis 41.

### Statistics

The ANOVA assay and Tukey post hoc test were conducted using JMP 12 software (SAS Institute) for most experiments except for western blotting (Student's t-test). In multi-comparison, JMP gave low-case letters for different treatments. The statistical difference between treatments was judged by non-overlapped lower-case letters (*p* < 0.05). Data were derived from triplicated experiments and are represented as the means ± SDs.

## Results

### Synergistic antiproliferative effects of cisplatin/PLB

**Figure 1:**
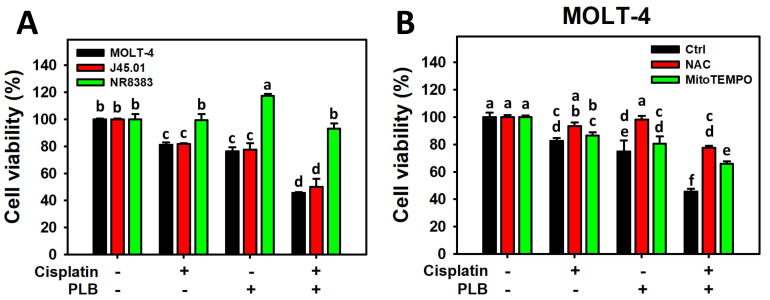
Cell viability for cisplatin and/or PLB treatments. Except for NAC or MitoTEMPO pretreatment or not, the cells were treated with the control (0.1% DMSO), cisplatin (3 μM), PLB (0.4 μM), and cisplatin/PLB (3 μM/0.4 μM) for 24 h, and their viabilities were analyzed by ATP assay. The data are marked at the top with lower-case letters, and nonoverlapping notes indicate statistical differences in multi-comparisons (*p* < 0.05).

Cisplatin/PLB, the combined treatment, showed cell viability of 45.51% and 49.94% in MOLT-4 and J45.01 cells, respectively, lower than cisplatin (81.07% and 81.79%) or PLB (76.36% and 77.40%) (Figure 1A). In comparison, the normal cell line (NR8383) showed limited cytotoxicity. The synergy index (α value) of cisplatin/PLB for the MOLT-4 and J45.01 cells was 1.36 and 1.27. This result revealed that cisplatin/PLB synergistically inhibited the proliferation of ALL cells. Since the MOLT-4 cells had a higher synergy index (α value) than J45.01 cells showed similar antiproliferative effects for cisplatin/PLB (1.36 and 1.27), MOLT-4 cells were chosen for the following experiments.

The ROS and MitoSOX inhibitors (NAC and MitoTEMPO) pretreatments were conducted before cisplatin or/and PLB treatments on MOLT-4 cells to assess the participation of cellular and mitochondrial oxidative stress. NAC and MitoTEMPO rescued the cisplatin/PLB-inhibiting proliferation (Figure 1B). Consequently, the results suggest ROS and MitoSOX contribute to the synergistic antiproliferative effect of cisplatin/PLB on MOLT-4 cells, although pharmacological inhibitor studies alone do not establish direct causality.

### Enhanced ROS and MitoSOX generation of cisplatin/PLB

As mentioned above, the role of cellular and mitochondrial oxidative stress (ROS and MitoSOX) on the synergistic antiproliferation of MOLT-4 cells treated with cisplatin/PLB was validated by their inhibitors such as NAC and MitoTEMPO, respectively (Figure 1B). However, oxidative stress, such as ROS and MitoSOX, was not assessed.

**Figure 2:**
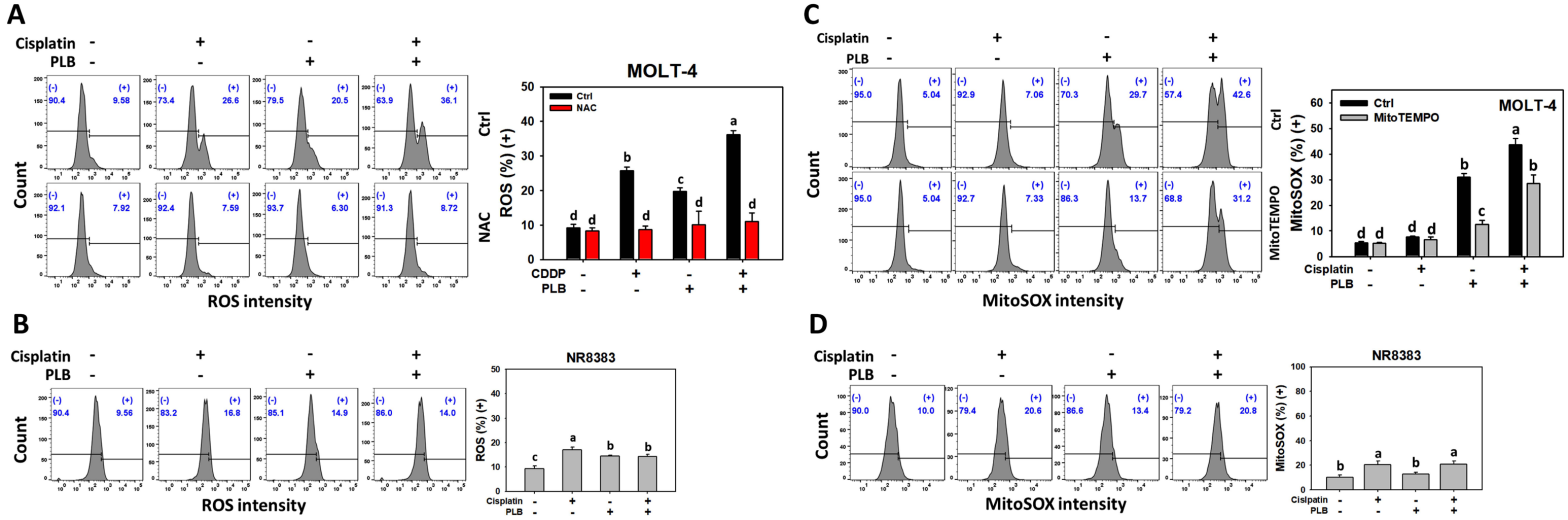
ROS and MitoSOX assays for cisplatin and/or PLB treatments. (A,C) ROS and MitoSOX levels of MOLT-4 cells. Except for NAC or MitoTEMPO pretreatment or not, MOLT-4 cells were treated with the control (0.1% DMSO), cisplatin (3 μM), PLB (0.4 μM), and cisplatin/PLB (3 μM/0.4 μM) for 24 h. (B,D) ROS and MitoSOX levels of normal cells (NR8383). Cells were treated in the same condition as (A,C), but no inhibitor was used. The ROS and MitoSOX-positive proportions (%) are labeled with (+) (%). The data are marked at the top with lower-case letters, and different letters indicate statistical differences in multi-comparisons (*p* < 0.05). Gating for ROS and MitoSOX produced similar proportions of positive events in the control, NAC, and MitoTEMPO groups under PLB(-)/cisplatin(-) conditions; the same gate positions were applied to all other treatments.

The ROS and MitoSOX response of cisplatin/PLB of MOLT-4 cells was determined via flow cytometry in parallel with normal cells (NR8383). Cisplatin/PLB caused a more significant ROS and MitoSOX proportion (+) (%) in MOLT-4 cells than in cisplatin, PLB, or the control (Figures 2A, 2C). In comparison, normal cells (NR8383) showed limited ROS and MitoSOX response in the cisplatin and/or PLB treatments (Figures 2B,2D).

NAC and MitoTEMPO pretreatments were conducted before cisplatin or/and PLB treatments on MOLT-4 cells to further confirm the cellular and mitochondrial oxidative stress status. NAC and MitoTEMPO rescued the cisplatin or/and PLB-induced ROS and MitoSOX increment (Figures 2A,2C). Consequently, cisplatin/PLB demonstrated the enhanced burst effects of ROS and MitoSOX on MOLT-4 cells.

### Enhanced MMP and GSH depletion of cisplatin/PLB

**Figure 3:**
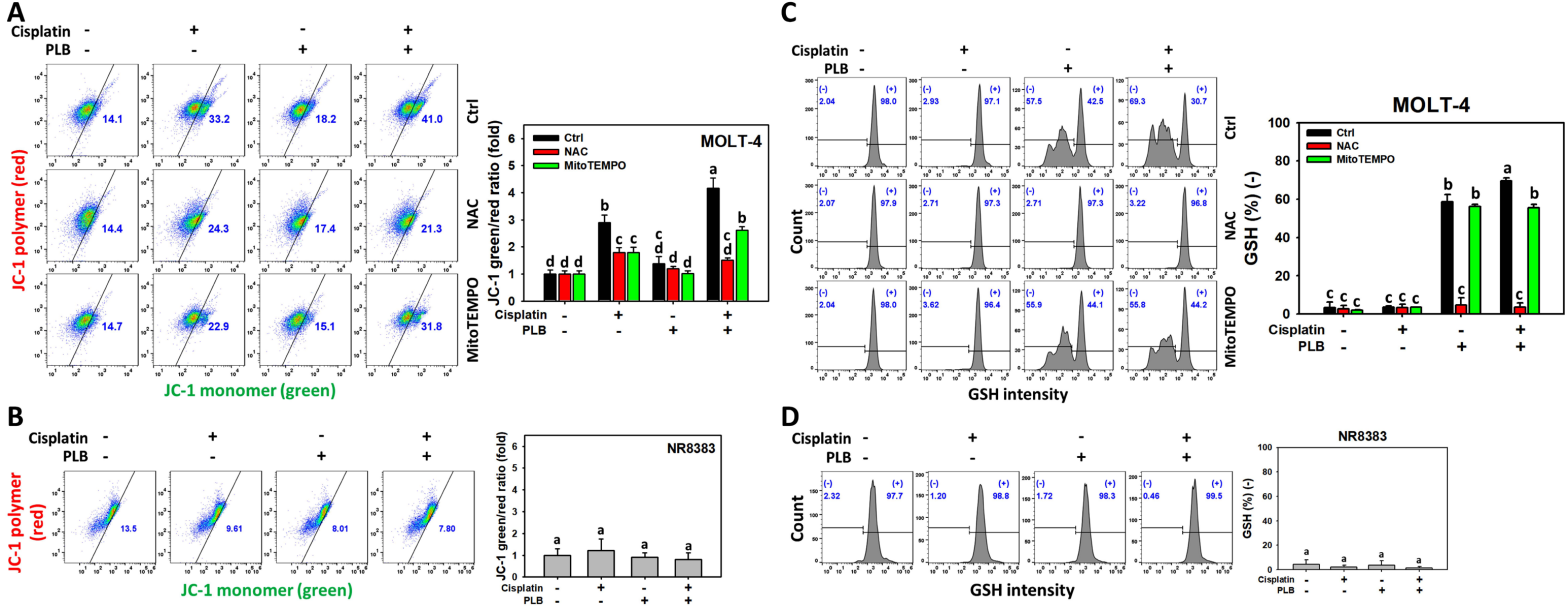
MMP and GSH assays for cisplatin and/or PLB treatments. (A,C) MMP and GSH levels of MOLT-4 cells. Except for NAC or MitoTEMPO pretreatment or not, MOLT-4 cells were treated with the control (0.1% DMSO), cisplatin (3 μM), PLB (0.4 μM), and cisplatin/PLB (3 μM/0.4 μM) for 24 h. (B,D) MMP and GSH levels of normal cells (NR8383). Cells were treated in the same condition as (A,C), but no inhibitor was used. The MMP status was evaluated by the JC-1 green/red ratio, where a high ratio indicated MMP depletion. The GSH-negative proportion (%) is labeled with (-) (%), where a high (-) (%) indicates GSH depletion. The data are marked at the top with lower-case letters, and different letters indicate statistical differences in multi-comparisons (*p* < 0.05). Gating for MMP and GSH produced similar proportions of positive events in the control, NAC, and MitoTEMPO groups under PLB(-)/cisplatin(-) conditions; the same gate positions were applied to all other treatments.

In addition to ROS and MitoSOX burst, MMP depletion is another sign of oxidative stress 44. The MMP response of cisplatin/PLB was determined via flow cytometry. Cisplatin/PLB exhibited a higher ratio (JC-1 green/red) in MOLT-4 cells than in cisplatin, PLB, or the control (Figure 3A), indicating MMP depletion. In comparison, normal cells (NR8383) showed limited MMP response in cisplatin and/or PLB treatments (Figure 3B).

NAC and MitoTEMPO pretreatments were conducted before cisplatin or/and PLB treatments on MOLT-4 cells to further confirm the oxidative stress status. NAC and MitoTEMPO rescued the cisplatin or/and PLB-induced MMP depletion, as evidenced by decreasing the ratio (JC-1 green/red) (Figure 3A). Consequently, the results suggest ROS and MitoSOX contribute to the enhanced MMP depletion of cisplatin/PLB on MOLT-4 cells, although pharmacological inhibitor studies alone do not establish direct causality.

GSH can counteract oxidative stress and the GSH level is associated with the modulation of oxidative stress 45. The GSH response of cisplatin/PLB was determined via flow cytometry. Cisplatin/PLB caused a more significant GSH proportion (-) (%) in MOLT-4 cells than in cisplatin, PLB, or the control (Figure 3C). In comparison, normal cells (NR8383) showed limited GSH response in cisplatin and/or PLB treatments (Figure 3D).

To further confirm the GSH status, NAC and MitoTEMPO pretreatments were conducted before cisplatin or/and PLB treatments on MOLT-4 cells. NAC and MitoTEMPO rescued the cisplatin or/and PLB-induced GSH depletion (Figure 3C). Consequently, the results suggest ROS and MitoSOX contribute to the enhanced GSH depletion of cisplatin/PLB on MOLT-4 cells, although pharmacological inhibitor studies alone do not establish direct causality.

### Enhanced subG1 increment of cisplatin/PLB

**Figure 4:**
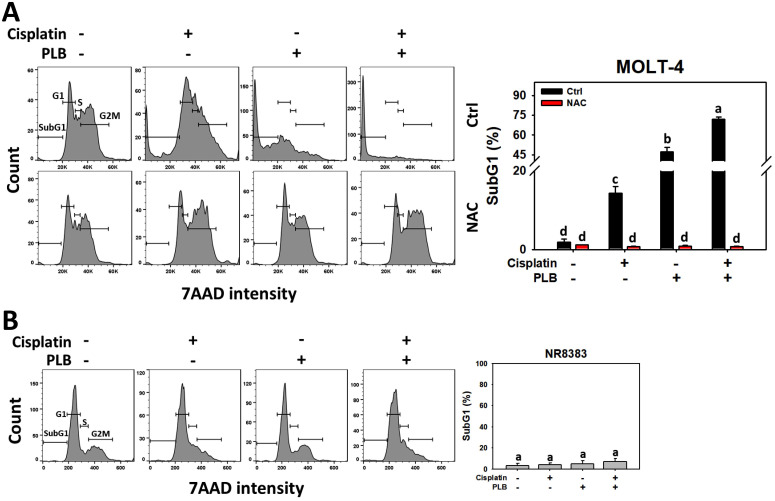
Cell cycle assay for cisplatin and/or PLB treatments. (A) SubG1 proportion of MOLT-4 cells. Except for NAC pretreatment or not, MOLT-4 cells were treated with the control (0.1% DMSO), cisplatin (3 μM), PLB (0.4 μM), and cisplatin/PLB (3 μM/0.4 μM) for 24 h. (B) SubG1 proportion of normal cells (NR8383). Cells were treated in the same condition as (A), but no inhibitor was used. The data are marked at the top with lower-case letters, and different letters indicate statistical differences in multi-comparisons (*p* < 0.05). Gating for G1 and G2/M was based on DNA content (2n and 4n, respectively); S phase was defined as the region between G1 and G2/M, and sub-G1 as events with DNA content below the G1 peak.

The participation of cell cycle interference in the synergistic antiproliferative effects of cisplatin/PLB was assessed (Figure 4A). Cisplatin/PLB increased the subG1 proportion (%) of MOLT-4 cells more than cisplatin, PLB, or the control. In comparison, normal cells (NR8383) showed limited subG1 response to the cisplatin and/or PLB treatments (Figure 4B). This subG1 phenomenon is an apoptosis-like indicator and warrants careful investigation.

Global cellular ROS has a more critical and direct role in broader cell cycle progression 46 than mitochondrial ROS alone 47. To assess the participation of global oxidative stress in cell cycle progression, NAC but not MitoTEMPO was introduced for pretreatment in the treatment of cisplatin or/and PLB on MOLT-4 cells. NAC rescued the cisplatin or/and PLB-induced subG1 increment (Figure 4A). Consequently, the results suggest ROS contributes to the enhanced subG1 accumulative effects of cisplatin/PLB on MOLT-4 cells, although pharmacological inhibitor studies alone do not establish direct causality.

### Enhanced apoptosis (annexin V) increment of cisplatin/PLB

**Figure 5:**
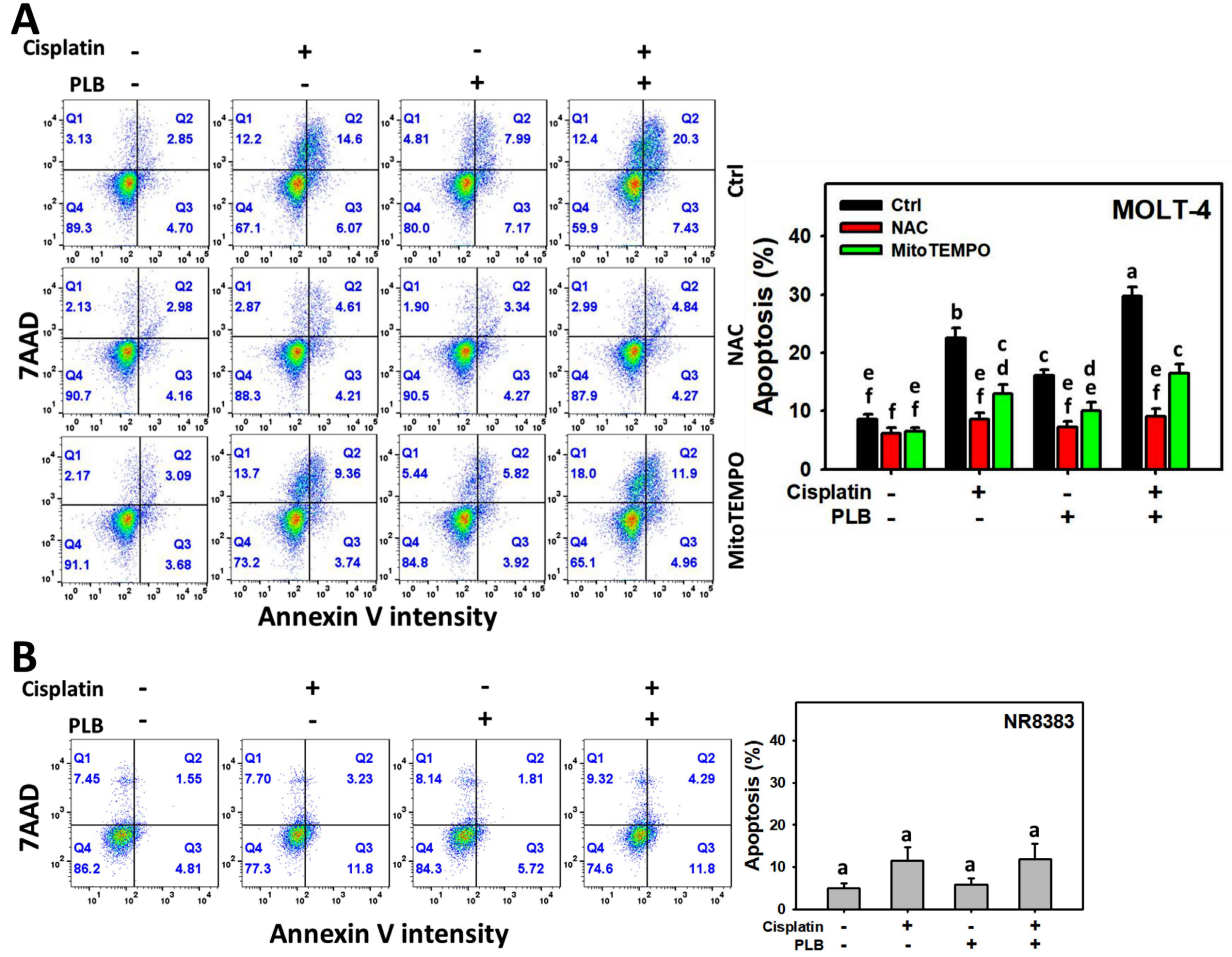
Annexin V/7AAD assay for cisplatin and/or PLB treatments. (A) Annexin V levels of MOLT-4 cells. Except for NAC or MitoTEMPO pretreatment or not, MOLT-4 cells were treated with the control (0.1% DMSO), cisplatin (3 μM), PLB (0.4 μM), and cisplatin/PLB (3 μM/0.4 μM) for 24 h. (B) Annexin V level of normal cells (NR8383). Cells were treated in the same condition as (A), but no inhibitor was used. The annexin V-positive proportion (%) is counted as apoptosis (%). The data are marked at the top with lower-case letters, and different letters indicate statistical differences in multi-comparisons (*p* < 0.05). Gating for annexin V produced similar proportions of positive events (Q2+Q3) in the control, NAC, and MitoTEMPO groups under PLB(-)/cisplatin(-) conditions; the same gate positions were applied to all other treatments.

The potential for apoptosis of MOLT-4 cells in the treatments of cisplatin or/and PLB was evaluated using the annexin V/7AAD method. Cisplatin/PLB caused more significant apoptosis (%) in MOLT-4 cells than in cisplatin, PLB, or the control (Figure 5A). Notably, its quadrant Q2 (late apoptosis) was higher than Q3 (early apoptosis). In comparison, normal cells (NR8383) showed limited apoptosis response to the cisplatin and/or PLB treatments (Figure 5B), which dominantly showed the early apoptosis (Q3). This suggests that cisplatin/PLB exerts more severe apoptosis in leukemia cells than normal cells.

To assess the participation of oxidative stress in apoptosis, NAC and MitoTEMPO pretreatments were conducted before cisplatin or/and PLB treatments on MOLT-4 cells. NAC and MitoTEMPO rescued the cisplatin or/and PLB-induced annexin V increment, i.e., apoptosis (Figure 5A). Consequently, the results suggest ROS and MitoSOX contribute to the enhanced apoptotic effects of cisplatin/PLB on MOLT-4 cells, although pharmacological inhibitor studies alone do not establish direct causality.

### Enhanced activation for intrinsic apoptotic signaling of cisplatin/PLB

**Figure 6:**
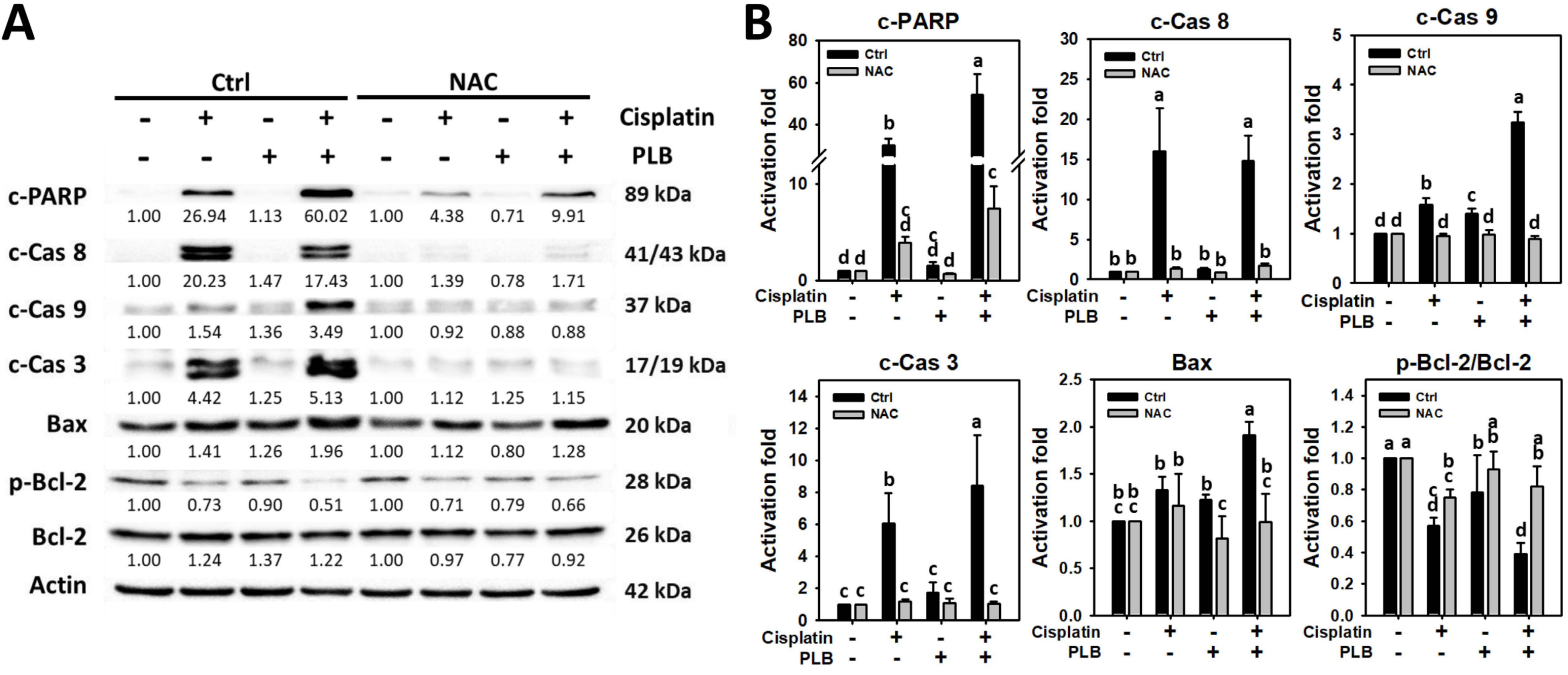
Apoptotic signaling assays. Except for NAC pretreatment or not, MOLT-4 cells were treated with the control (0.1% DMSO), cisplatin (3 μM), PLB (0.4 μM), and cisplatin/PLB (3 μM/0.4 μM) for 24 h, and their apoptotic signaling status was analyzed via Western blotting. The data are marked at the top with lower-case letters, and different letters indicate statistical differences in multi-comparisons (*p* < 0.05). p-Bcl-2 (Ser70) is used as anti-apoptotic marker.

PARP, the DNA repair enzyme, is cleaved by activated caspase 3 and is regarded as a hallmark of apoptosis 48. To assess the involvement of extrinsic and intrinsic caspases, the levels of cleaved caspases 8 and 9 were examined. Cisplatin/PLB caused higher expressions of cleaved caspases 3 and 9 in MOLT-4 cells than in cisplatin, PLB, or the control (Figure 6). However, the extrinsic caspase 8 levels were non-significantly different between cisplatin/PLB and cisplatin alone, although they were higher than the control and PLB alone.

To further assess the role of intrinsic caspases, the levels of caspase 9's upstream proapoptotic and anti-apoptotic proteins, such as Bax and Bcl-2, were examined 49. When the p-Bcl-2/Bcl-2 ratio is larger than 1, it indicates that cells were triggered to intrinsic apoptosis 50. Consistently, cisplatin/PLB upregulated higher Bax in MOLT-4 cells than in other treatments. In contrast, cisplatin/PLB downregulated the p-Bcl-2 (anti-apoptotic Ser70)/Bcl-2 ratio more than the single treatments (Figure 6).

Since Figure 5A showed that NAC is more effective in suppressing apoptosis (as indicated by annexin V), we therefore take advantage of this to use NAC to investigate its apoptotic signaling without MitoTEMPO. To assess the participation of cellular oxidative stress in PARP cleavage and caspases 8/9/3 activation, NAC pretreatment was conducted before cisplatin or/and PLB treatments on MOLT-4 cells. NAC rescued the cisplatin or/and PLB-induced apoptotic signaling (Figure 6). Consequently, the results suggest ROS contributes to the enhanced intrinsic apoptotic signaling of cisplatin/PLB on MOLT-4 cells, although pharmacological inhibitor studies alone do not establish direct causality.

### Enhanced γH2AX and 8-OHdG increment of cisplatin/PLB

**Figure 7:**
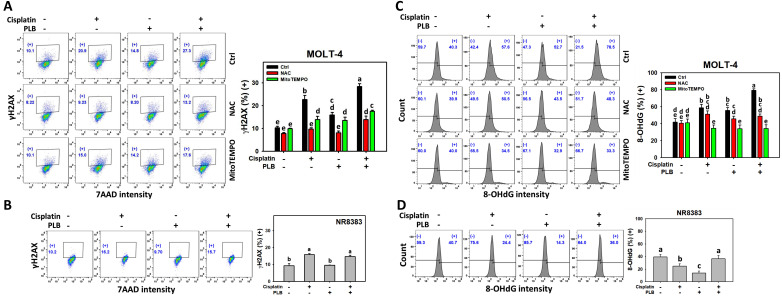
γH2AX and 8-OHdG assays for cisplatin and/or PLB treatments. (A,C) γH2AX and 8-OHdG levels of MOLT-4 cells. Except for NAC or MitoTEMPO pretreatment or not, MOLT-4 cells were treated with the control (0.1% DMSO), cisplatin (3 μM), PLB (0.4 μM), and cisplatin/PLB (3 μM/0.4 μM) for 24 h. (B,D) γH2AX and 8-OHdG levels of normal cells (NR8383). Cells were treated in the same condition as (A,C), but no inhibitor was used. The γH2AX and 8-OHdG-positive proportion (%) is labeled with (+) (%). The data are marked at the top with lower-case letters, and different letters indicate statistical differences in multi-comparisons (*p* < 0.05). Gating for γH2AX and 8-OHdG produced similar proportions of positive events in the control, NAC, and MitoTEMPO groups under PLB(-)/cisplatin(-) conditions; the same gate positions were applied to all other treatments.

The γH2AX and 8-OHdG response of cisplatin/PLB was determined via flow cytometry. Cisplatin/PLB caused a more significant γH2AX and 8-OHdG proportion (+) (%) in MOLT-4 cells than in cisplatin, PLB, or the control (Figures 7A,7C). In comparison, normal cells (NR8383) showed limited γH2AX and 8-OHdG response to the cisplatin and/or PLB treatments (Figures 7B,7D).

To further confirm the DNA damage status, NAC and MitoTEMPO pretreatments were conducted before cisplatin or/and PLB treatment on MOLT-4 cells. NAC and MitoTEMPO rescued the cisplatin or/and PLB-induced γH2AX and 8-OHdG increment (Figures 7A,7C). Consequently, the results suggest ROS and MitoSOX contribute to the enhanced γH2AX and 8-OHdG DNA damage of cisplatin/PLB on MOLT-4 cells, although pharmacological inhibitor studies alone do not establish direct causality.

## Discussion

In this investigation, we assessed the proliferation-regulating effects of the combined treatment of cisplatin and PLB in leukemia cells. Mechanisms underlying cell cycle, oxidative stress, apoptosis, and DNA damage induced by cisplatin/PLB were explored and compared with those induced by cisplatin or PLB alone.

Natural products have been applied to sensitize the chemotherapy responses for cancer cells 51, 52. For example, ethyl acetate *Nepenthes* extract (EANV)/cisplatin combined treatment shows the synergistic antiproliferation of oral cancer cells 51. *Artocarpus heterophyllus*-derived artocarpanone and cycloartocarpin combined with cisplatin synergistically suppress lung and breast cancer cell proliferation 52.

Several literature reports have explored the potential reasons for the cisplatin-sensitizing effects of natural products. Both cisplatin and several natural products exhibit ROS-modulating potential that may converge to promote this, potentially leading to the antiproliferation of cancer cells. For example, low-dose cisplatin upregulates oxidative stress-responsive enzyme activity (superoxide dismutase and catalase) and lipid peroxidation 53 of leukemia (HL-60) cells. Similarly, *Lithospermum erythrorhizon*-derived shikonin 54 enhance cisplatin-induced ROS generation in colon cancer cells. Therefore, the synergistic antiproliferation effects of combined treatment (cisplatin/natural products) are partly attributed to their enhanced generation of oxidative stress.

Similarly, the examples of cisplatin/PLB combined treatment show the synergistic antiproliferative effects of cisplatin/PLB on several types of cancer cells, such as cervical 25 and tongue 26. However, cisplatin/PLB for leukemia cells has not yet been reported. The enhanced ROS generation of cisplatin/PLB has been reported in tongue cancer cells 26, improving the antiproliferative effect. Although PLB exhibits anti-leukemia effects 27, 55, its combined effects with cisplatin have been rarely investigated. In the present investigation, the antiproliferative effects of cisplatin/PLB in leukemia cells (MOLT-4 and J45.01) were validated synergistically (synergy index = 1.36 and 1.27) (Figure 1A). Because the cervical study 25 used a 48 h MTT assay and the tongue study 26 used a 24 h CCK-8 assay, their cisplatin/plumbagin results are not directly comparable with our leukemia data obtained by a 24 h ATP assay. However, these findings suggest that the cisplatin/PLB combination could be a promising anticancer therapy. PLB shows good drug safety in normal peripheral blood mononuclear cells 55 and animal studies 28. Similarly, cisplatin/PLB exhibits non-cytotoxicity in normal cells (Figure 1A). Therefore, cisplatin/PLB offers anti-leukemia functions without significant side effects to normal cells. Given that the cervical 25, tongue 26, and our leukemia experiments were all *in vitro*, conclusions about therapeutic promise and minimal toxicity remain tentative; *in vivo* validation is necessary to advance the cisplatin/PLB combination toward clinical translation.

These synergistic antiproliferative effects of cisplatin/PLB in leukemia cell treatment were attenuated by NAC and MitoTEMPO (Figure 1B). This raises the possibility that cellular and mitochondrial oxidative stress play a vital role in the anti-leukemia effects. It warrants a deep assessment of cellular and mitochondrial oxidative stress in cisplatin/PLB. Notably, cisplatin/PLB induces greater cellular and mitochondrial oxidative stress via ROS and a MitoSOX burst than either cisplatin or PLB alone (Figure 2), as confirmed by their respective inhibitors. Consequently, the impacts of cellular and mitochondrial oxidative stress were confirmed in the present study. Notably, cisplatin primarily increases cellular ROS, whereas PLB induces both cellular ROS and mitochondrial MitoSOX in leukemia cells (Figure 2). NAC pretreatment reduces cisplatin-induced cytotoxicity in tongue cancer cells 56, implying that ROS contributes to cisplatin efficacy. Therefore, PLB-driven ROS—including cellular and mitochondrial ROS—may amplify cisplatin's activity and underlie the synergistic response observed in MOLT-4 cells. Moreover, the antioxidant Nrf2/HO-1 pathway is frequently upregulated in malignancies (such as cisplatin-resistant A549 lung cancer cells 57) and contributes to apoptosis evasion. Therefore, assessing how the cisplatin/PLB combination affects Nrf2 and HO-1 expression in MOLT-4 cells should be investigated in future studies.

This enhanced oxidative stress of leukemia cells was also validated by downregulating MMP (Figure 3A), an oxidative stress indicator 44. Since oxidative stress is counteracted by cellular antioxidants such as GSH 58, the downregulation of GSH appropriately causes oxidative stress generation. Consistently, cisplatin/PLB shows higher GSH (-) proportions in leukemia cells than cisplatin or PLB (Figure 3C), indicating that GSH depletion is highly induced by cisplatin/PLB. Hence, the enhanced GSH depletion may contribute to the enhanced oxidative stress exerted by cisplatin/PLB. Moreover, both cisplatin/PLB-induced MMP and GSH depletion in leukemia cells were attenuated by NAC and MitoTEMPO, suggesting that cisplatin/PLB enhances cellular and mitochondrial oxidative stress in leukemia cells.

Oxidative stress may stimulate apoptosis 59 and DNA damage 60 in cancer cells. These oxidative stress responses are prone to trigger high levels of apoptosis and DNA damage. Cisplatin upregulates oxidative stress, DNA damage 53, and apoptosis 61 in leukemia (HL-60) cells. Several examples of combined treatment for cisplatin and anticancer drugs are provided as follows. For instance, the nitrated 6,6,6 tricycle derivative (SK2)/cisplatin 62 induce high apoptosis and DNA damage due to increased oxidative stress in oral cancer cells. Shikonin synergistically induces oxidative stress to promote cisplatin-induced apoptosis in colon cancer cells 54. Thymoquinone promotes cisplatin-triggered apoptosis and oxidative DNA damage (γH2AX and 8-OHdG) of osteosarcoma cells, accompanied by synergistically inducing ROS 62. Similarly, cisplatin/PLB triggers more subG1 accumulation (Figure 4), annexin V-detected apoptosis (Figure 5), and DNA damage (γH2AX and 8-OHdG) (Figure 7) in leukemia cells than single treatments. Furthermore, both cisplatin/PLB-induced γH2AX and 8-OHdG expression in leukemia cells were attenuated by NAC and MitoTEMPO, suggesting that cisplatin/PLB enhances DNA damage in leukemia cells via oxidative stress.

Moreover, higher intrinsic caspase 9 and the apoptosis executioner caspase 3 were activated by cisplatin/PLB than by single treatments (Figure 6), accompanied by enhanced oxidative stress in leukemia cells. This caspase 9 activation in cisplatin/PLB was also supported by the upregulated Bax and downregulated Bcl-2 activation (p-Bcl-2 (Ser70, anti-apoptotic 43)/Bcl-2 ratio). As shown in Figure 6, cisplatin/PLB reduced Bcl-2 phosphorylation at Ser70, an anti-apoptotic marker, and this decrease was reversed by NAC, indicating that Ser70 dephosphorylation of p-Bcl-2 is driven by oxidative stress from the cisplatin/PLB combination. Because AKT (protein kinase B) upregulates Bcl-2 expression 63, AKT may be downregulated by cisplatin/PLB, causing apoptosis. Moreover, NAC attenuated the activation of caspase 3 and 9 in cisplatin/PLB. These results suggest cellular oxidative stress triggers intrinsic caspase signaling and apoptosis. Oxidative stress can inhibit AKT signaling in Chinese herb rhubarb-derived rhein-triggered apoptosis of pancreatic cancer cells 64. Since oxidative stress was upregulated in cisplatin/PLB, it warrants a deep investigation of the role of AKT in the synergistic regulation of antiproliferation in cisplatin/PLB.

PLB has been investigated as a chemotherapy sensitizer. In addition to leukemia cells, in a phase I trial, PLB improved the efficacy of androgen deprivation therapy in prostate cancer 65. PLB also enhances cisplatin sensitivity in gastric cancer cells 66 and potentiates tamoxifen-induced apoptosis in breast cancer cells 67. Together, these findings support PLB as a broad chemosensitizer that can increase the effectiveness of diverse anticancer therapies.

There are some limitations in the current study. Although NAC and MitoTEMPO are widely used to implicate oxidative stress, they have pleiotropic effects beyond ROS and MitoSOX scavenging; therefore, complementary analyses of antioxidant substrates and enzymes are needed to avoid over-attributing causality to ROS and MitoSOX in NAC and MitoTEMPO rescue experiments. Because NR8383 rat lung macrophages were the only normal control, the observed selectivity may be model-specific; using human normal hematopoietic cells or peripheral blood mononuclear cells, as well as animal models, in follow-up experiments would substantially bolster translational relevance. Moreover, another limitation of the present study is that it investigated optimal conditions for combined PLB/cisplatin treatment but measured only cell viability at fixed doses rather than determining IC_50_ values. The Chou-Talalay method for drug combination synergy analysis relies on the median effect equation, in which the median effect dose D_m_ corresponds to the drug's IC_50_ derived from a dose-response curve. Because IC_50_ values were not available, the Chou-Talalay method could not be applied and was therefore not used in this study. Overall, this limitation restricts the study by assessing interactions at specific fixed doses, potentially overlooking effects that change with doses.

## Conclusions

The cisplatin/PLB combined treatment has not been reported previously in leukemia cells. This study validates the synergistic and selective antiproliferative activity and mechanisms of cisplatin/PLB against leukemia cells *in vitro*, with limited cytotoxicity to the normal NR8383 macrophage model. Mechanistic experiments implicate combined cellular and mitochondrial oxidative stress driving apoptosis and DNA damage, and antioxidant probes (NAC and MitoTEMPO) modulate these effects. These results support the therapeutic potential of cisplatin/PLB but remain preliminary because they are based on in vitro assays and a single normal cell comparator.

## Figures and Tables

**Figure 8:**
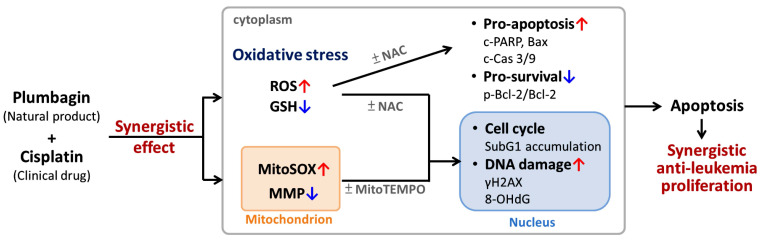
Overview of the effects and mechanisms of cisplatin/PLB synergistic anti-leukemia proliferation. As shown in Figures 1-7, all cisplatin/PLB-induced synergistic effects in Figure 8 were higher in leukemia cells than in normal macrophages (not shown in this figure). NAC and MitoTEMPO are inhibitors of cellular and mitochondrial oxidative stress. Mechanistically, the synergistic antiproliferative effect of cisplatin/PLB on MOLT-4 cells was attenuated by NAC and MitoTEMPO. The enhanced oxidative stresses were confirmed, because the induction of ROS/MitoSOX and the depletion of MMP/GSH of cisplatin/PLB were more highly regulated in leukemia cells than in normal macrophages. These oxidative stress responses were also attenuated by NAC and/or MitoTEMPO, suggesting a potential role for enhanced oxidative stress in the synergistic antiproliferative effect of cisplatin/PLB treatment in leukemia cells. Moreover, these enhanced oxidative stresses caused by cisplatin/PLB were validated as triggering apoptosis (subG1 and annexin V results) and intrinsic apoptotic signaling, as evidenced by NAC, as well as triggering DNA damage (γH2AX and 8-OHdG), as evidenced by NAC and MitoTEMPO. Notably, these cisplatin/PLB mechanisms exhibited higher performance in leukemia cells than in normal macrophages.

## Data Availability

Data will be made available on request.
